# Taxonomic notes on the genus *Campiglossa* Rondani (Diptera, Tephritidae, Tephritinae, Tephritini) in India, with description of three new species

**DOI:** 10.3897/zookeys.977.57875

**Published:** 2020-10-22

**Authors:** Karamankodu Jacob David, David Lawrence Hancock, Santhamma Salini, Ramasamy Gandhi Gracy, Kandiyil Sachin

**Affiliations:** 1 National Bureau of Agricultural Insect Resources, Bangalore-560024, Karnataka, India National Bureau of Agricultural Insect Resources Bangalore India; 2 60 South Street, Carlisle, Cumbria CA1 2EP, UK Unafiliated Cumbria United Kingdom

**Keywords:** Asteraceae, *
Conyza
*, *
Dioxyna
*, northeast India, *
Sonchus
*, Western Ghats

## Abstract

Three new species of *Campiglossa* Rondani are described from India: adults of both sexes and third instar larvae of *C. ialong* David, Salini & Hancock, **sp. nov.** and *C. sherlyae* David & Hancock, **sp. nov.**, plus an adult female of *C. shaktii* David, Sachin & Hancock, **sp. nov.**, are described and illustrated. Postabdominal structures, cephalopharyngeal skeleton, and anterior and posterior spiracles of *C. gemma* (Hering, 1939) and *C. sororcula* (Wiedemann, 1830) are illustrated. DNA barcode sequences of *C. ialong***sp. nov.**, *C. sherlyae***sp. nov.**, and *C. gemma* were obtained and reported. Records of *C. absinthii* (Fabricius, 1805) and *C. iracunda* (Hering, 1938) are regarded as misidentifications of *C. lyncea* (Bezzi, 1913) and *C. shaktii***sp. nov.**, respectively, and excluded from the Indian fauna. A key to the known species of *Campiglossa* from India is provided. Results of preliminary phylogenetic analysis using COI revealed that *C. ialong***sp. nov.** is paraphyletic to the *Campiglossa misella* group and C. *C. sherlyae***sp. nov.** is a sister species of *C. deserta*.

## Introduction

*Campiglossa* Rondani is one of the most speciose genera in the subfamily Tephritinae with nearly 200 described species (Norrbom et al. 1999; Han and Ro 2019). They are characterised by an elongate proboscis, a predominantly spinulose preglans area of the phallus, and bases of the antennae widely separated by a space 0.5–1 times the width of the scape (Korneyev 1999). *Campiglossa* is predominantly a Palaearctic genus but has representatives in other zoogeographic regions. Most species are associated with host plants of the family Asteraceae. The Afrotropical fauna was revised by Munro (1957) and the Palaearctic fauna by Korneyev (1990) and Merz (1992). Han and Ro (2019) synonymised *Homoeotricha* Hering and *Dioxyna* Frey with *Campiglossa* based on their analysis employing the mtCOI marker, but study of related genera is required before precise generic limits can be determined. The Indian fauna was studied by Bezzi (1913), Agarwal et al. (1989), and Hancock and McGuire (2002). Although Agarwal and Sueyoshi (2005) listed eight species of *Campiglossa* from India, Hancock (2008) regarded report of *C. iracunda* (Hering, 1938) from India as a misidentification, while record of *C. absinthii* (Fabricius, 1805) is also regarded as a misidentification, as discussed below. Three new species of *Campiglossa* encountered in India during surveys for fruit flies are described here. Postabdominal structures and larvae of *C. gemma* (Hering, 1939) and *C. sororcula* (Wiedemann, 1830) from southern India are described and illustrated along with taxonomic notes on the four other recorded Indian species. As types of these four species were not available for study, detailed redescriptions or diagnoses are not included.

## Material and methods

Specimens deposited in NBAIR were examined for the study. Following are the acronyms used in the text:

**NBAIR** ICAR – National Bureau of Agricultural Insect Resources, Bangalore, India

**NPC** National Pusa Collection, Indian Agricultural Research Institute, New Delhi, India

**ZSI** Zoological Survey of India, Kolkata, India

Collections were made by sweep netting and rearing infested flowers of host plants belonging to family Asteraceae. Images of specimens were taken using a Leica DFC 420 camera mounted on a Leica M205A stereozoom microscope; images of genitalia were taken using an 8 MP camera temporarily attached to a Leica DM 1000 compound research microscope. Multiple images were stacked and combined to a single image using Combine ZP (Hadley 2011). Line drawings were made using a drawing tube attached to a Leica DM 1000 compound microscope. Measurements of male and female genitalia were taken using Leica Automontage Software, LAS 3.4. Terminology adopted here follows White et al. (1999). Singular form is used for all paired organs and setae in the text (e.g., one postpronotal lobe seta means one pair of postpronotal lobe setae). Ratios have been calculated as per Han and Ro (2019).

### DNA isolation and partial gene sequencing of COI

To isolate the genomic DNA, the hind and mid legs (one each) of individual insects were used and the DNA isolation was carried out using the Qiagen DNeasy Blood & Tissue Kit method following the manufacturer’s protocol. After obtaining the DNA, the quality and quantity were estimated using nanodrop-BioRad. PCR amplification of partial gene sequences of mitochondrial COI gene was carried out by using the universal COI primers (Hebert et al. 2004). PCR amplification was performed for a total volume of 30 μL, containing 2 μl DNA extract (20 ng), 1 μl (2mol) of each primer, 1 μl dNTP mixture (2.5 mmol for each), 2.5 μL 10x Taq PCR reaction buffer, 3 μL 25 mM MgCl_2_^+^, and 1 unit of Taq DNA polymerase using a thermal cycler (BioRad iCycler) with the PCR cycle as follows: initial step at 94 °C for 1 minute and 35 cycles of the following: denaturing 95 °C for 30 seconds, annealing 51 °C for 30 seconds, extension at 72 °C for 45 seconds and 4 °C thereafter (Ball and Armstrong 2008). The PCR products size varied from 650 to 680 bp; the amplified products were confirmed by running on 1.5% agarose gel with 250 bp ladder and visualized in INGENIUS gel dock. The amplified products were purified using Qiagen PCR purification Kit by following the manufacturer’s instructions and the purified samples were sequenced using Sanger’s method. The sequences were annotated using NCBI Blast tools and submitted to NCBI GenBank Database where accession numbers were obtained (*C. ialong* sp. nov. – MT169786; *C. sherlyae* sp. nov. – MT019895; *C. gemma* – MT169785; *C. sororcula* – MT019889)

### Construction of molecular phylogeny tree

The molecular phylogeny of *Campiglossa* was constructed using the software MEGAX (Kumar et al. 2018). A total of 18 DNA barcode sequences were used for this analysis including the outgroup *Tephritis conura* Loew, in which four were from India and another 14 were downloaded from NCBI database. *Campiglossa* from Oriental, Palaearctic, and Nearctic regions were included in the analysis. The evolutionary relationship was inferred using the maximum likelihood method. The General Time Reversible model (Nei and Kumar 2000) was used with uniform rate of substitution. The bootstrap consensus tree inferred from 1000 replicates was taken to represent the evolutionary history of the taxa analyzed (Felsenstein 1985). Branches corresponding to partitions reproduced in less than 50% bootstrap replicates were collapsed. Initial tree(s) for the heuristic search were obtained automatically by applying the maximum parsimony method. This analysis involved 18 nucleotide sequences. Codon positions included were 1^st^ + 2^nd^ + 3^rd^.

## Results

### Key to species of *Campiglossa* Rondani from India

**Table:** 

1	Scutellum with one pair of distinct setae, the apical pair absent or vestigial; wing pattern reticulate with dark markings pale and diffuse	***C. sororcula* (Wiedemann)**
–	Scutellum with two pairs of setae, the apicals distinct; wing with dark markings distinct	**2**
2	All femora yellow or yellowish orange with no trace of brown or black colour	**3**
–	All femora predominantly black/brown	**5**
3	Apex of cell r_4+5_ without a hyaline spot, apical scutellar seta as long as basal, spermatheca elongate and tubular, aculeus tip broad with preapical indentations	***C. gemma* (Hering)**
–	Apex of cell r_4+5_ with a hyaline spot, apical scutellar seta shorter than basal, spermatheca oval or round, aculeus tip pointed with or without preapical indentation	**4**
4	Pterostigma with two yellow or hyaline spots, aculeus tip with preapical indentation, spermathecae round	***C. shaktii* David, Sachin & Hancock, sp. nov.**
–	Pterostigma with a single hyaline spot, aculeus tip pointed without preapical indentation, spermathecae oval	***C. ialong* David, Salini & Hancock, sp. nov.**
5	Posterior notopleural seta black; cell r_2+3_ with one hyaline marginal spot	**6**
–	Posterior notopleural seta white; cell r_2+3_ with two hyaline marginal spots	**7**
6	Base of the cell r_2+3_ in wing usually with three round hyaline spots before the crossvein r-m (distribution: Kashmir)	***C. producta* (Loew)**
–	Base of the cell r_2+3_ in wing predominantly black or brown with single prominent hyaline spot near the crossvein r-m (distribution: southern India)	***C. sherlyae* David & Hancock, sp. nov.**
7	Wing with hyaline discal spots between apices of veins R_1_ and Cu_1_ large and often crossing cells; pterostigma with a single, medial hyaline spot	***C. lyncea* (Bezzi)**
–	Wing with hyaline discal spots between veins R_1_ and Cu_1_ small and rounded, not crossing cells; pterostigma with two (small or large) hyaline spots	**8**
8	Hyaline spots in pterostigma very small and rounded; abdomen with two submedian yellow spots each on tergites 1+2 to 6, scutum with longitudinal stripes	***C. kumaonensis* Agarwal et al.**
–	Hyaline spots in pterostigma large and quadrate; abdomen with two median black spots on each abdominal tergite except tergite 1+2; scutum without longitudinal stripes	***C. cribellata* Bezzi**

### Taxonomy

#### Genus Campiglossa

Taxon classificationAnimaliaDipteraTephritidae

Rondani

EFB8E78F-3601-5C66-ACAD-40DA29932B7A

Campiglossa Rondani, 1870: 121. Type species Tephritis irrorata Fallen, by original designation.

##### Diagnosis.

antennae widely separated by 0.5–1× width of scape; proboscis elongate and geniculate; scutum with dorsocentral setae placed near transverse suture; posterior notopleural seta black, brown, yellow, or white; apical scutellar seta often shorter than basals; epandrium elongate and usually with a lateral surstylar flange; preglans area of phallus spinulose; glans of phallus with elongate tubular acrophallus; aculeus tip often with preapical indentations; spermatheca tubular, oval or round, and spinulose (Korneyev 1990; Merz 1994).

#### Campiglossa ialong

Taxon classificationAnimaliaDipteraTephritidae

David, Salini & Hancock sp. nov.

7D1EBAA0-16AE-5337-9B63-D98274AE1D63

http://zoobank.org/ECA22E62-C83C-458E-8CC7-9830831F6E99

Figures 1 , 2–9 , 10–11

##### Diagnosis.

Medium-sized fly (3.74–4.25 mm), body grey, pollinose, with white setulae; scutum without prominent stripes; abdomen with submedian black markings; wing with reticulate pattern.

**Figure 1.**
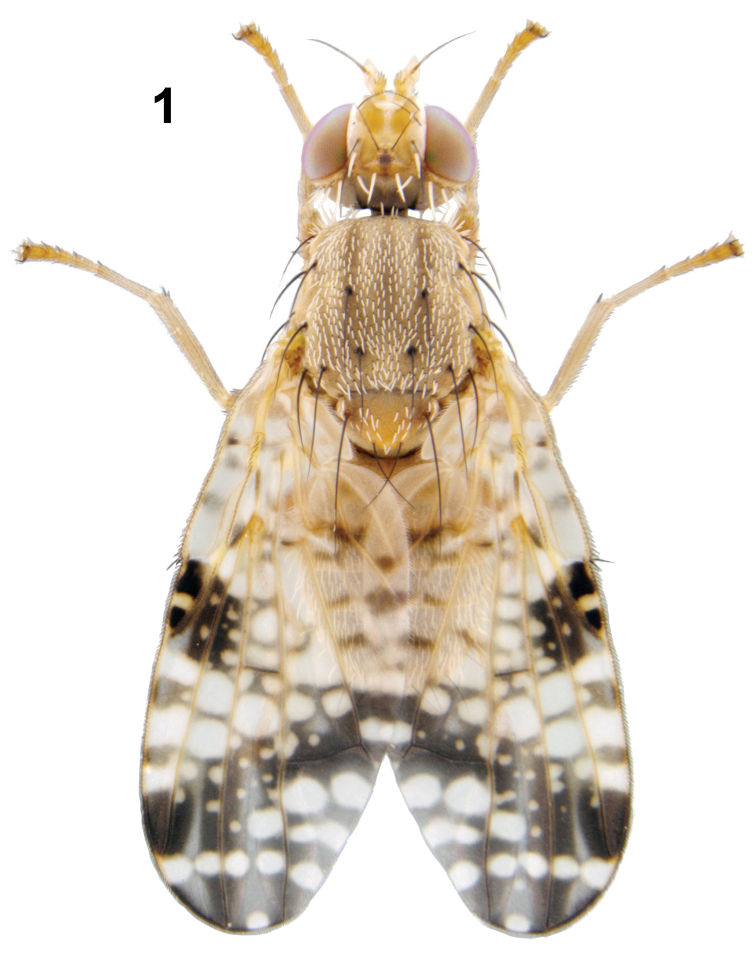
Habitus (male) of *Campiglossa ialong* David, Salini & Hancock, sp. nov.

##### Description.

Male (body length, 3.74–4.25 mm; wing length, 3.76–4.04 mm).

**Figures 2–9.**
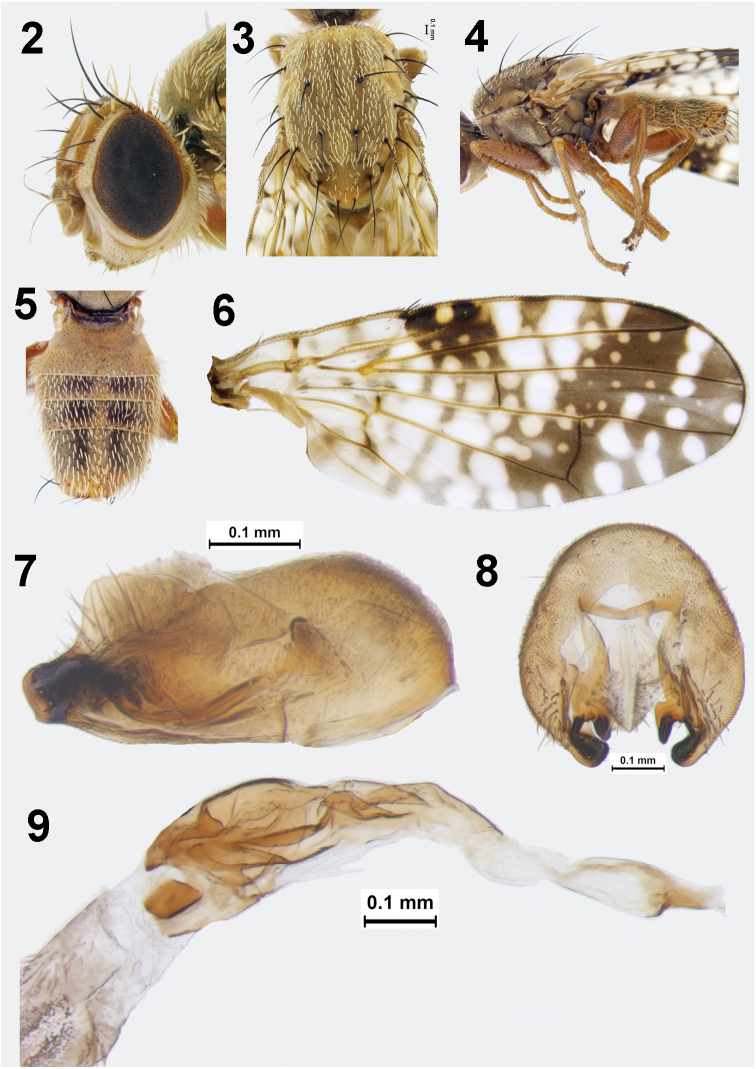
*Campiglossa ialong* David, Salini & Hancock, sp. nov. **2** head **3** thorax (dorsal view) **4** thorax (lateral view) and legs **5** abdomen **6** wing **7** epandrium and surstyli (lateral view) **8** epandrium and surstyli (posterior view) **9** glans of phallus.

***Head***: Slightly higher than long (head ratio 0.83–0.86); frons fulvous (frons-head ratio 0.38–0.40), with a medial band of pruinosity from ocellar triangle to lunule leaving two dark fuscous lateral bands devoid of pruinosity; two frontal setae (three in a few specimens); two subequal orbital setae (posterior one white); well-developed proclinate ocellar seta (0.7 length of medial vertical seta); lateral vertical seta white; medial vertical seta black; paravertical seta white; postocular setae intermixed black and white. Scape, pedicel, and flagellomere concolorous with frons; pedicel plus flagellomere shorter than face; arista bare; face concave with raised epistomal margin; gena and occiput fulvous. Eye ratio 0.64–0.69; gena-eye ratio 0.13–0.18; antenna-head ratio 0.45–0.47; arista-antenna ratio 1.20–1.45.

***Thorax***: Scutum grey pollinose, with three faint stripes and well-developed chaetotaxy (all setae black); one postpronotal lobe seta, one presutural supra-alar seta, one anterior notopleural seta, one posterior notopleural seta, one dorsocentral seta near transverse suture, placed anterior of postsutural supra-alar seta and posterior notopleural seta, one presutural supra-alar seta, one postalar seta, one intra-alar seta, one prescutellar acrostichal seta. Anepisterum grey with single black anepisternal seta in line with posterior notopleural seta; anepisternum covered with tiny white setulae; thick white setulae posteriorly near phragma; anepimeron without any black setae, with thick stubby white setulae anteriorly; katepisternum with single black seta posterior to phragma in dorsal region; anatergite and katatergite grey without any setulae; haltere pale yellow. Scutellum flat, yellow with sparse white setulae; two scutellar setae; apical scutellar seta 2/3 length of basal scutellar seta. Mediotergite grey, without setulae.

***Legs***: All segments unicolorous, yellowish orange; fore femur with single row of five or six stout ventral setae, two rows of dorsal setae; mid and hind femur covered with tiny black setulae. Mid tibia with four apical spines, one elongate, the others all 1/4 length of prominent spine.

***Wing***: Reticulate pattern, with hyaline and yellow spots; basal 1/3 hyaline with faint brown markings; apical 2/3 dark brown with numerous hyaline and yellow spots. Cell bc hyaline; cell c hyaline with two faint brown markings; pterostigma dark brown with a medial, yellow spot/patch; apex of cell r_1_ and r_2+3_ black without any hyaline spots; cell r_2+3_ with a preapical dumbbell-shaped spot. Cell r_1_ with three broad hyaline patches and irregular yellow spots or patches; cell r_2+3_ hyaline only in basal portion, rest brown to black with irregular yellow spots and, broad hyaline markings that are extensions of the hyaline markings from cell r_1_ and preapical dumbbell-shaped spot (separate spots in a few specimens). Cell br predominantly hyaline, with irregular brown markings; cell r_4+5_ predominantly black or brown with an apical hyaline spot, two preapical spots, numerous yellow spots, and a broad basal hyaline spot. Cells bm and bcu hyaline; basal 2/3 of cell dm largely hyaline, with narrow basal and submedial brown transverse bands, apical 1/3 brown with hyaline spots; cell m with four broad irregular markings; cell cu_2_ and anal lobe predominantly hyaline with irregular brown markings.

***Abdomen***: Grey pollinose, with white setulae; tergites 1+2 broad, with reduced pruinosity; tergites 3–5 with broad, submedian, quadrate patches; tergite 5 is 2–2.25× broader than tergites 3 and 4, with apical black setae. Sternites grey; posterior margin of sternite 5 with shallow concavity.

***Male genitalia***: Epandrium elongate, tapering towards surstylar end (lateral view) without clear demarcation between surstylus and epandrium. Lateral surstylar flange as high as epandrium, serrate throughout its entire length; apex of lateral surstylus without clear demarcation of anterior and posterior lobes; proctiger hyaline, microtrichose. Epandrium oval in outline (caudal view); medial surstylus well developed with prensisetae. Phallus elongate (1.34 mm); preglans area strongly spinulose; basal lobe absent; glans of phallus sclerotised, 1/2 length of phallus (0.78 mm), with well-developed, elongate, tubular acrophallus.

***Female***: Similar to male except larger (body length 4.56–5.23 mm; wing length 4.14–4.62 mm). Oviscape shining black (1.66 mm); taeniae short (0.25 of total length of eversible membrane); spicules on anterior end of eversible membrane (1.30 mm) conical with pointed apex, whereas spicules of distal end conical with blunt apex. Aculeus elongate (1.38 mm) with pointed tip, devoid of preapical indentations. Spermatheca dark brown, oval, with transverse striations.

**Figures 10, 11.**
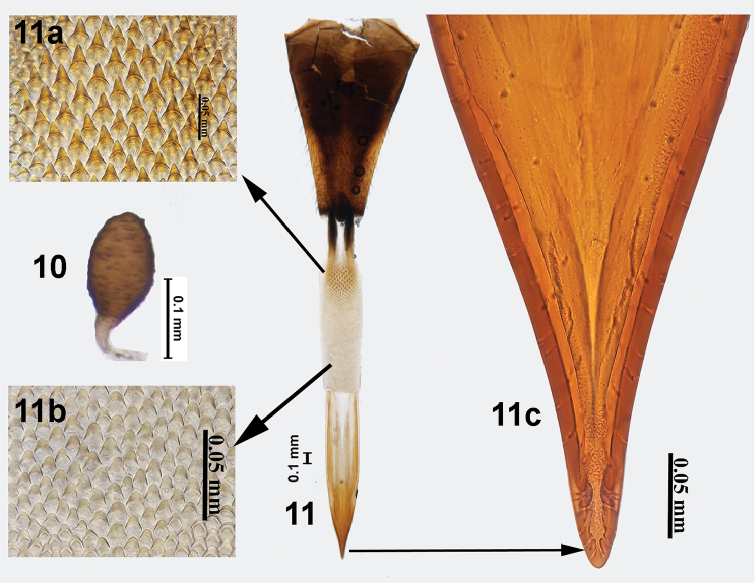
*Campiglossa ialong* David, Salini & Hancock, sp. nov. **10** spermatheca **11** ovipositor **11a** spicules on proximal end of eversible membrane **11b** spicules on distal end of eversible membrane **11c** aculeus tip.

##### Type material.

Holotype ♂, INDIA: Meghalaya, Mihmyntdu, Ialong, 25.476°N, 92.226°E, 13.x.2019, Salini S. Paratypes: 21♂♂, 7♀♀, same data as above except for two males with collector's name David K.J. 1 larva on slide (III instar), same data as above (NBAIR).

##### DNA barcode.

GenBank accession number MT169786 (1♂, INDIA: Meghalaya, Mihmyntdu, Ialong, 25.476°N, 92.226°E, 24.x.2019, K.J. David).

##### Etymology.

The specific epithet is a noun in apposition and refers to the type locality.

##### Third instar larva (Figs 12–14).

Larva short, stout (3.22–3.51 mm), whitish to dull white. Mouthhook pointed with a well-developed preapical tooth as long as apical mouthhook; ventral apodeme 2× broader than mouthhook; mandibular neck not prominent; dorsal apodeme pointed dorsally; labial sclerite elongate; pharyngeal sclerite 2.5× longer than broad; hypopharyngeal bridge reduced; parastomal bar prominent; dorsal bridge pointed anteriorly; ventral bridge of hypopharyngeal sclerite pointed anteriorly; anterior sclerite not well developed; dorsal cornua undivided; ventral cornua with two branches. Anterior spiracle weakly sclerotised, with six tubules. Posterior spiracle with spiracular slits oval, slightly longer than wide, devoid of transverse striations; spiracles separated by distance equal to the length of each slit; dorsal and ventral spiracular bundle with 2–6 single hairs; lateral spiracular bundle with 4–6 single hairs.

**Figures 12–14.**
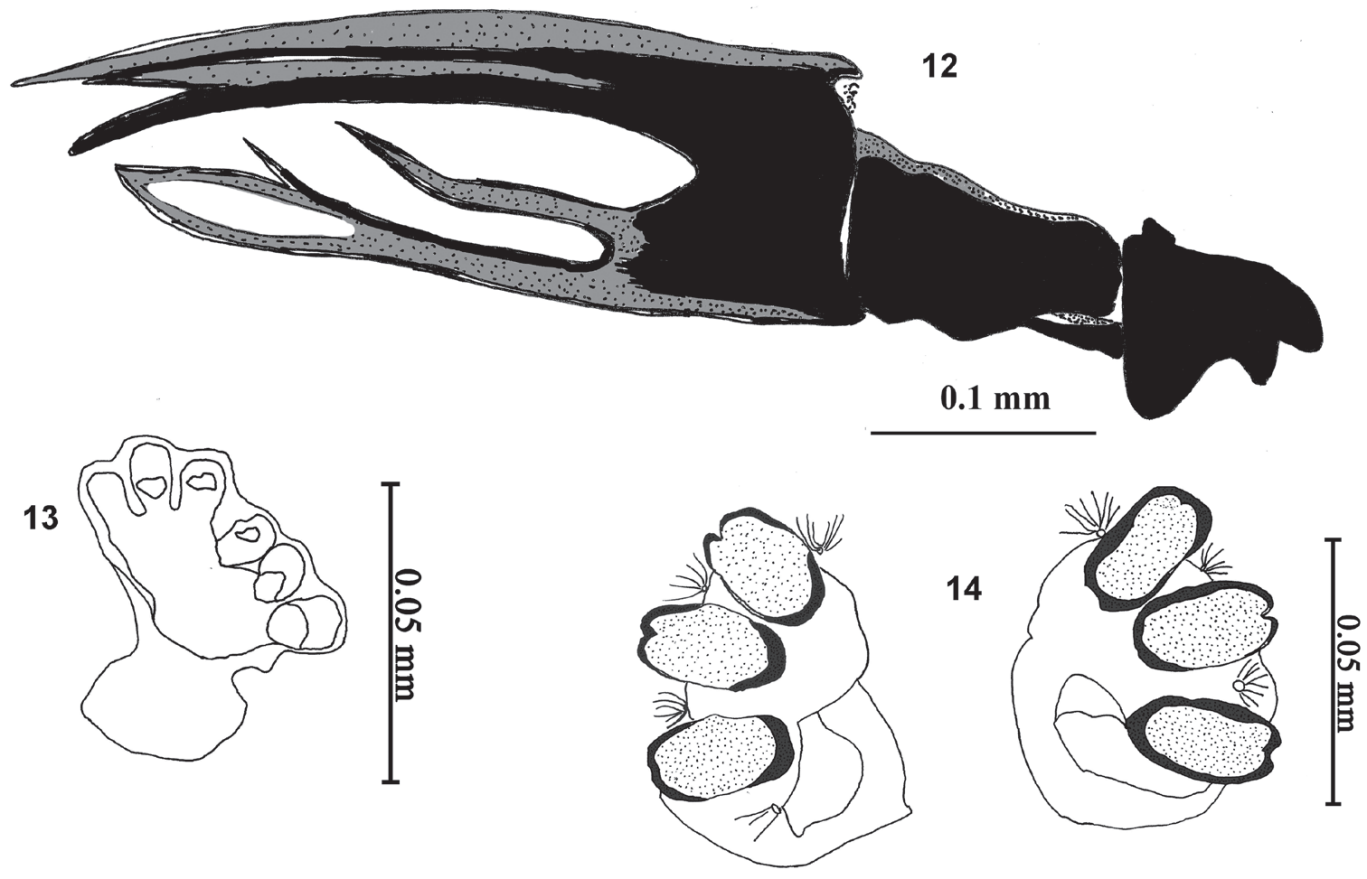
Larval morphology of *Campiglossa ialong* David, Salini & Hancock, sp. nov. **12** cephalopharyngeal skeleton **13** anterior spiracle **14** posterior spiracles.

##### Remarks.

*Campiglossa ialong* is most similar to *C. iracunda* (Hering) in appearance but with only one hyaline spot at the apex of cell R_2+3_, as in *C. siamensis* (Hardy 1973). However, the black posterior notopleural seta differs from *C. siamensis*, which has a brown or yellowish seta. As per the phylogenetic tree (Fig. 51), it is paraphyletic with the *misella* group.

#### Campiglossa shaktii

Taxon classificationAnimaliaDipteraTephritidae

David, Sachin & Hancock sp. nov.

797CB314-579D-5586-811F-CF0BC66EAF2D

http://zoobank.org/BC902BFB-A5BE-45F1-9543-F2C66F81391D

Figures 15–19 , 20–22

##### Diagnosis.

Medium-sized fly (4.42–4.85 mm), body predominantly grey pollinose, with white setulae; scutum without prominent stripes; abdomen uniformly grey without submedian black markings; wing with reticulate pattern.

**Figures 15–19.**
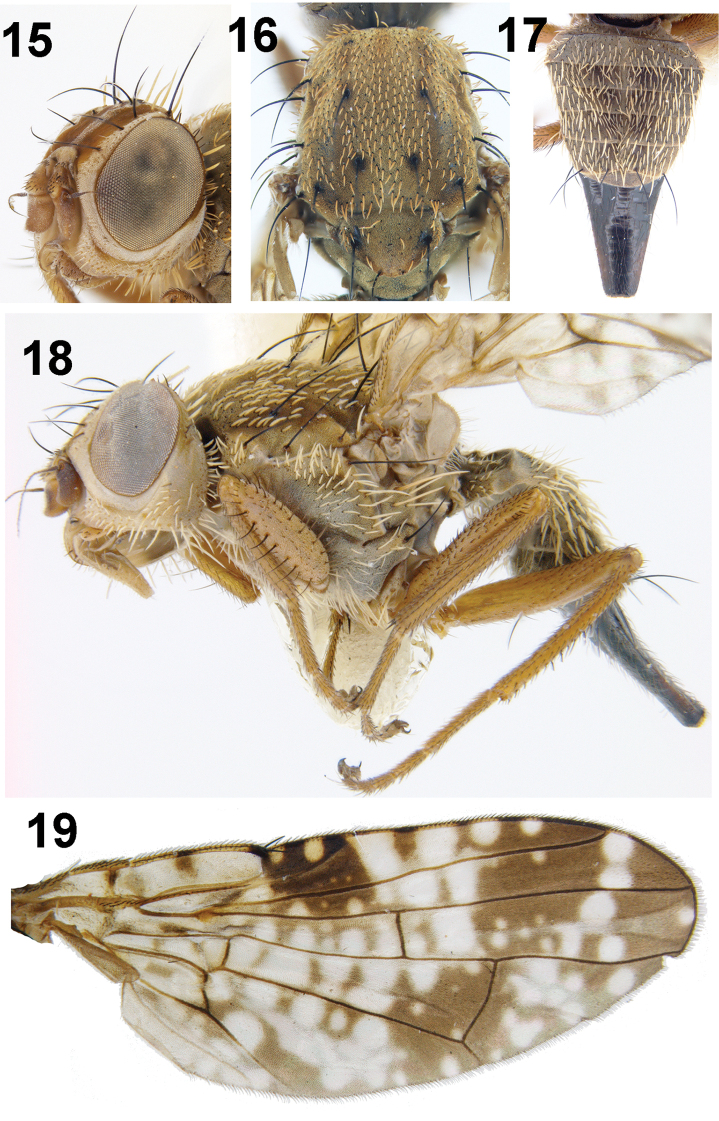
*Campiglossa shaktii* David, Sachin & Hancock, sp. nov. **15** head **16** thorax (dorsal view) **17** abdomen **18** thorax (lateral view) and legs **19** wing.

##### Description.

Female (body length 4.40–4.66 mm; wing length 4.30–4.70 mm).

**Figures 20–22.**
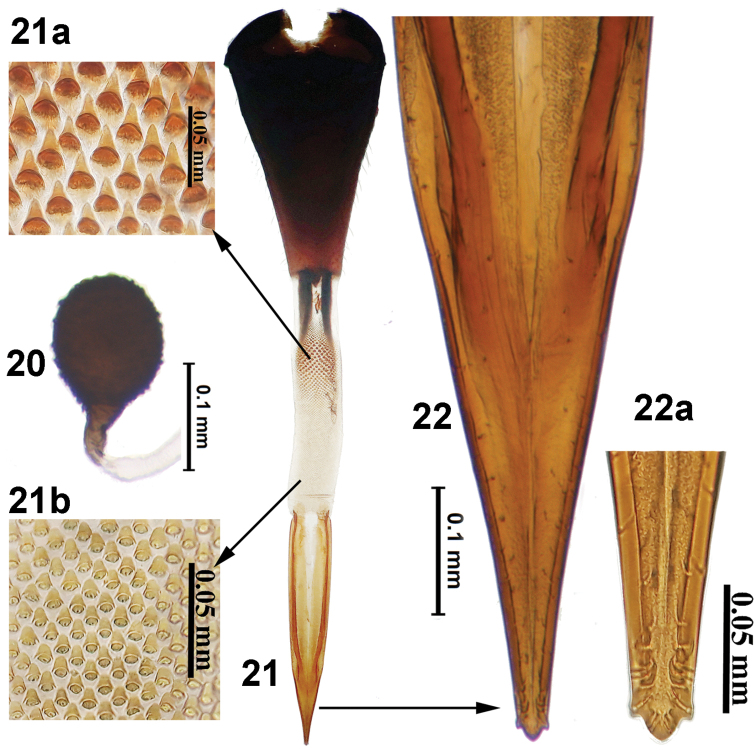
*Campiglossa shaktii* David, Sachin & Hancock, sp. nov. **20** spermatheca **21** ovipositor **21a** spicules on proximal end **21b** spicules on distal end of eversible membrane **22** aculeus **22a** aculeus tip.

***Head***: Slightly higher than long (head ratio 0.84–0.94), frons fulvous (frons-head ratio 0.42− 0.45), with a medial band of pruinosity from ocellar triangle to lunule leaving two dark fuscous lateral bands devoid of pruinosity; two frontal setae; two orbital setae; posterior one white, shorter than anterior; well-developed proclinate ocellar seta (0.7 length of medial vertical seta) longer than orbital and frontal setae; lateral vertical seta white; medial vertical seta black; paravertical white; postocular setae intermixed black and white. Scape, pedicel, and flagellomere concolorous with frons; pedicel plus flagellomere shorter than face; arista bare; face concave with raised epitsomal margin; gena and occiput fulvous. Eye ratio 0.65–0.72; gena-eye ratio 0.17–0.19; antenna-head ratio 0.39–0.42; arista-antenna ratio 1.34–1.49.

***Thorax***: Scutum grey pollinose with three faint stripes and well developed chaetotaxy (all setae black); one postpronotal lobe seta, one presutural supra-alar seta, one anterior notopleural seta, one posterior notopleural seta, one dorsocentral setae near transverse suture, placed anterior of postsutural supra-alar seta and posterior notopleural seta, one presutural supra-alar seta, one postalar seta, one intra-alar seta, one prescutellar acrostichal seta. Anepisternum grey, with a single black anepisternal seta in line with posterior notopleural seta; anepisternum covered with white setulae in posterior half; elongate setae near phragma; anepimeron without any black setae, with thick, stubby, white setulae anteriorly; katepisternum with single black setae posterior to phragma; anatergite and katatergite grey, without any setulae; haltere pale yellow. Scutellum flat, grey, with sparse, white setulae; two pairs of scutellar setae; apical scutellar seta 2/3 length of basal scutellar seta. Mediotergite grey, without setulae.

***Legs***: All segments unicolorous, yellowish orange; fore femur with single row of six or seven stout ventral setae, two rows of dorsal setae; mid and hind femur covered with tiny black setulae. Tibiae and tarsi with rows of spines; mid tibia with four apical spines, one elongate, the others all 1/4 length of prominent spine.

***Wing***: Reticulate pattern, with hyaline and yellow spots; cell bc hyaline with a brown spot on humeral crossvein; cell c hyaline with a single brown patch medially; pterostigma dark brown, with two round, yellow spots, the one closer to apex of vein Sc smaller compared to distal one; apex of cell r_1_ and r_2+3_ black, without any hyaline spots. Cell r_1_ with three broad, hyaline patches and irregular yellow spots; cell r_2+3_ dark basally, with two faint yellow spots or markings and with a preapical dumbbell-shaped spot. Cell br predominantly hyaline, with irregular brown markings; cell r_4+5_ predominantly black or brown, with a small apical hyaline spot, three preapical spots arranged in a triangle, numerous yellow spots, and hyaline basally. Cells bm and bcu hyaline; cell dm basally broadly hyaline with three narrow, transverse, brown bands to level of r-m crossvein; apically brown with hyaline spots; cell m with diffuse hyaline markings; cell cu_2_ and anal lobe predominantly hyaline with irregular brown markings.

***Abdomen***: Grey pollinose with white setulae; tergite 1 with reduced pruinosity; tergites grey without dark markings; oviscape glossy black and equal in length to tergites 4–6.

***Female genitalia***: Oviscape dark brown to black (1.59 mm); eversible membrane as long as oviscape, with taeniae short (0.3 mm); spicules on proximal end of eversible membrane (1.44 mm) conical, well sclerotised, whereas spicules at distal end broadly conical and weakly sclerotised. Aculeus tip trilobed, with preapical indentation. Spermatheca black, round, spinose.

##### Type material.

Holotype ♀, INDIA: Sikkim, Lachung, 08.vi.2012, Shakti K. Singh. Paratypes: 1♀, same data as holotype (NBAIR).

##### Etymology.

This species is named after its collector, Shakti Kumar Singh.

##### Remarks.

This species is undoubtedly the ‘*Paroxyna*’ or ‘Campiglossa’ iracunda of previous authors (Kapoor et al. 1979; Kapoor 1993; Agarwal and Sueyoshi 2005), the identity of which was discussed by Hancock (2008) and regarded as a misidentification.

#### Campiglossa sherlyae

Taxon classificationAnimaliaDipteraTephritidae

David & Hancock sp. nov.

67C29E78-9270-5FF2-84C3-1CDA9067A734

http://zoobank.org/A53ED0F8-DD7A-4A8F-A59F-B42B1F1C20C0

Figures 23 , 24–28

##### Diagnosis.

Small fly (male 2.50–2.90 mm; female 2.80–3.36 mm); body grey pollinose, without prominent stripes; abdomen grey with submedian black markings; wing with reticulate pattern.

**Figure 23.**
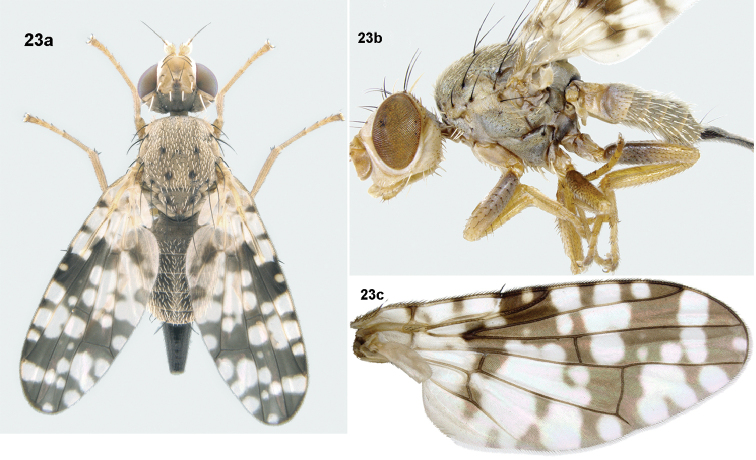
*Campiglossa sherlyae* David & Hancock, sp. nov. **23a** habitus (dorsal) **23b** habitus (lateral) **23c** wing.

**Figures 24–28.**
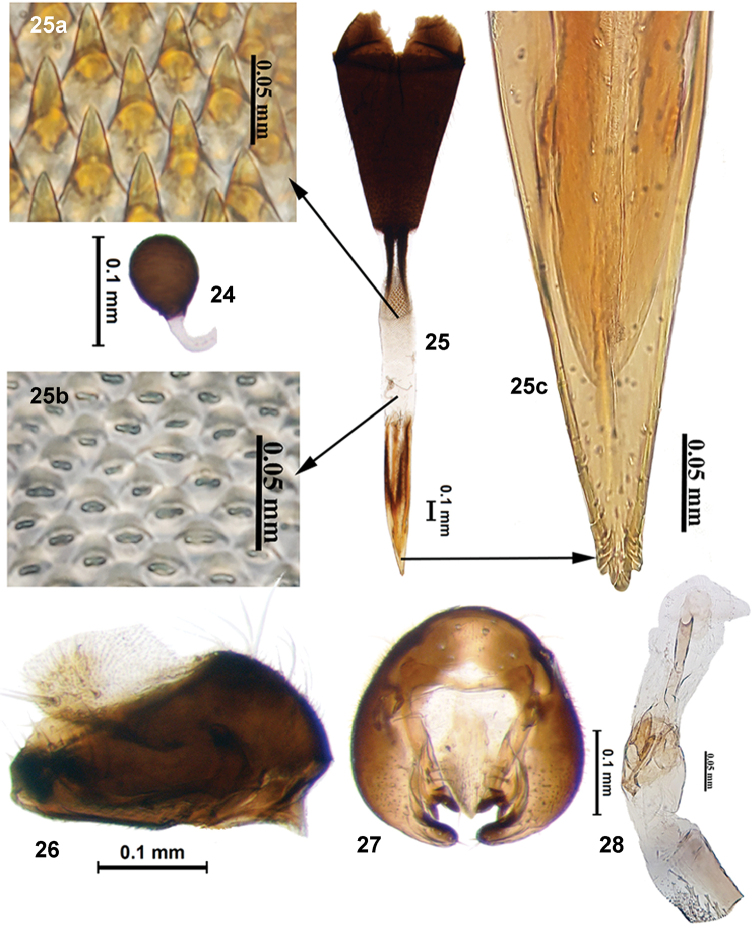
*Campiglossa sherlyae* David & Hancock, sp. nov. **24** spermatheca **25** ovipositor **25a** spicules on proximal end of eversible membrane **25b** spicules on distal end of eversible membrane **25c** aculeus tip **26** epandrium and surstyli (lateral view) **27** epandrium and surstyli (posterior view) **28** glans of phallus.

##### Description.

Female (body length 2.80–3.36 mm; wing length 2.50–3.00 mm).

***Head***: Nearly as long as high (head ratio 0.95–0.96), frons fulvous (frons-head ratio 0.40–0.41), with two frontal setae, two orbital setae (posterior orbital seta white), postocellar and postvertical seta white; lateral vertical seta white; medial vertical seta black; ocellar seta black and longer than frontal and orbital setae; postocular setae intermixed black and white. Scape, pedicel, and flagellomere concolorous with frons; pedicel plus flagellomere shorter than face; arista bare; face concave, with raised epistomal margin; gena and occiput fulvous. Eye ratio 0.70–0.79; gena-eye ratio 0.14–0.15; antenna-head ratio 0.44–0.50; arista-antenna ratio, 1.22–1.38.

***Thorax***: Scutum grey pollinose, without stripes and chaetotaxy well-developed (all setae black); one postpronotal lobe seta, one presutural supra-alar seta, one anterior notopleural seta, one posterior notopleural seta, one dorsocentral seta near transverse suture, placed anterior of postsutural supra-alar seta and posterior notopleural seta, one presutural supra-alar seta, one postalar seta, one intra-alar seta, one prescutellar acrostichal seta. Anepisterum grey, with single black anepisternal seta in line with posterior notopleural seta; anepisternum covered with white setulae; anepimeron without any black setae; katepisternum with single black seta posterior to phragma, anatergite, and katatergite grey without any setulae; haltere pale yellow. Scutellum flat, grey, with sparse white setulae; two scutellar setae; apical scutellar seta 1/2 length of basal scutellar seta. Mediotergite grey, without setulae.

***Legs***: All femora with extensive black markings (0.75 of all femora with black markings), all other segments fulvous; fore femur with single row of four or five stout ventral setae, two rows of eight or nine dorsal setae; mid and hind femur covered with tiny black setulae. Tibiae and tarsi with rows of spines; mid tibia with four subequal apical spines.

***Wing***: Reticulate pattern with hyaline and yellow spots; cell bc hyaline with a brown streak on humeral crossvein; cell c hyaline, with a single brown band medially; pterostigma dark brown, with a single hyaline spot, apex of cell r_1_ and r_2+3_ without hyaline spot. Cell r_1_ with three broad, hyaline patches, cell r_2+3_ with three broad, hyaline markings. Cell br hyaline basally and with a broad preapical hyaline patch; cell r_4+5_ with five uneven, hyaline spots (basal and subapical larger than medial and apical spot); apex of cell r_4+5_ with small hyaline spot. Cells bm and bcu hyaline; cell dm predominantly hyaline with base and apex brown; cell m with a broad, hyaline mark (formed by fusion of three spots) and a preapical spot; cell cu_2_ predominantly hyaline, with brown streaks and apical hyaline spot; apex of cell bcu with brown patch.

***Abdomen***: Grey pollinose, with white setulae. Tergite 1 with reduced pruinosity; tergites grey with submedian markings on tergites 3–6; oviscape black and equal in length to tergites 4–6.

***Male postabdomen***: Epandrium well sclerotised, without clear delineation between epandrium and lateral surstylus; proctiger hyaline, with densely arranged setae anteriorly; surstylar flange prominent, with serrated edge; epandrium and surstyli oval in outline in posterior view; medial surstylus with well-developed apical prensisetae. Phallus, excluding glans, 1.2 mm long; glans of phallus with well-developed tubular acrophallus.

***Female postabdomen***: Oviscape black (1.02 mm), not longer than the combined length of last three abdominal segments. Eversible membrane (0.85 mm) with well-developed taeniae; spicules on proximal end of eversible membrane elongate and conical; distal end with broad conical spicules. Aculeus (0.89 mm) with tip trilobed. Spermatheca round, brown, granulose.

##### Type material.

Holotype ♀, INDIA, Karnataka, Bangalore, Attur, 23.ix.2013, David, K. J. Paratypes: 3♂♂, 1♀, INDIA, Karnataka, Bangalore, Attur, 23.ix.2013, David K.J.; 4♂♂, 4♀♀, INDIA, Karnataka, Tumkur, Kunigal, 04.xii.2013, David K.J.; 1♂, INDIA, Karnataka, Bangalore, Attur, 09.xii.2013, David K.J.; 1♀, INDIA, Karnataka, Bangalore, Hebbal, 02.i.2014, David K.J.; 1♂, 1♀, INDIA, Karnataka, Bangalore, Attur, 08.xii.2014, Prabhu G.; 1♂, 2♀♀, INDIA, Karnataka, Bangalore, Attur, 13.x.2016, Prabhu G.; 1♂, 1♀, INDIA, Karnataka, Bangalore, Attur, 16.x.2016, Prabhu G.; 1♀, INDIA, Karnataka, Tumkur, Devarayanadurga, 04.iv.2017, Prabhu G.; 1♀, INDIA, Karnataka, Bangalore, Attur, 12.xii.2017, Prabhu G.; 1 larva in slide (III instar): INDIA: Karnataka, Bangalore, Attur, 18.xi.2013, Prabhu G. (NBAIR).

##### DNA barcode.

NCBI GenBank accession number MT019895 (1♂, INDIA: Karnataka, Bangalore, Attur, 03.ix.2019, Sachin, K.).

##### Etymology.

The species is named after the late Sherly Joseph, in memory of the first author’s sister.

##### Third instar larva (Figs 29–31).

Larva short (3.88–4.14 mm), fusiform, creamy white. Mouthhook pointed with a well-developed preapical tooth as long as the apical mouthhook; ventral apodeme broader than mouthook; mandibular neck not prominent; dorsal apodeme pointed dorsally, conical; labial sclerite elongate; hypopharyngeal sclerite longer than broad; hypopharyngeal bridge reduced; parastomal bar reaching midway of hypopharyngeal sclerite; ventral bridge of hypopharyngeal sclerite pointed anteriorly; anterior sclerite present; dorsal cornua undivided; ventral cornua with two branches. Anterior spiracle weakly sclerotised with six tubules. Posterior spiracle with spiracular slits oval, slightly longer than wide, devoid of transverse striations; spiracles separated by a distance twice the length of each slit; dorsal and ventral spiracular bundle absent; lateral spiracular bundle with three single hairs.

**Figures 29–31.**
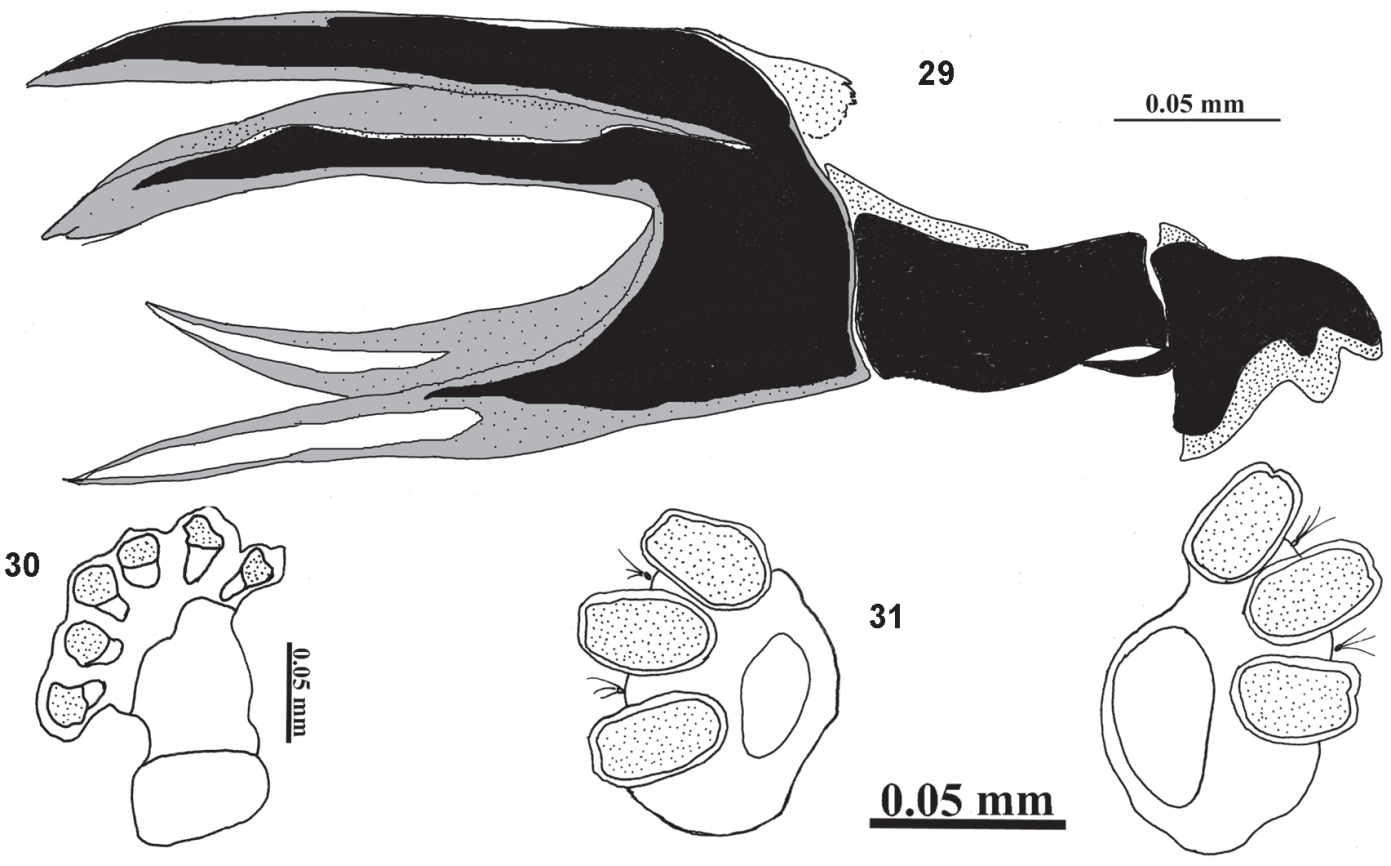
III instar larva of *Campiglossa sherlyae* David & Hancock, sp.nov. (Hering) **29** cephalopharyngeal skeleton **30** anterior spiracle **31** posterior spiracles.

##### Host plant.

Flowers of *Sonchus* sp. (Asteraceae).

##### Remarks.

This species belongs in the *producta* group and is known only from Karnataka. It was misidentified as *C. deserta* (Hering, 1939) by Hancock and McGuire (2002) and their Indian record of a female from Mudigere, Karnataka, is *C. sherlyae*. Other records listed by Hancock and McGuire (2002) from Thailand and Vietnam appear to have been properly identified as *C. deserta*, which is a species widespread in China (including Guangxi Province), Korea, and Japan. *Campiglossa sherlyae* is very similar to *C. producta* and *C. deserta*, differing from *C. producta* in possessing predominantly black or brown base of cell r_2+3_ in wing with a prominent spot near crossvein r-m, and from *C. deserta* in lacking a hyaline base to cell r_2+3_ and in having *Sonchus* rather than *Lactuca* as its host plant. The phylogenetic tree (Fig. 51) shows that this species and Korean samples of *C. deserta* are closely related but with a 2% divergence based on a NCBI-GenBank sequence similarity search (BLAST), along with differences in morphological characters and host plant, suggest they are distinct.

#### Notes on other Indian species

##### Campiglossa gemma

Taxon classificationAnimaliaDipteraTephritidae

(Hering, 1939)

53B0F4E8-76CD-5C76-BB78-2424F9281121

Figures 32–37

Paroxyna gemma Hering, 1939: 183. Type locality: Kodaikanal, Tamil Nadu, India.

###### Material examined.

10♂♂, 4♀♀, INDIA, Tamil Nadu, HRS Kodaikanal, 01.iv.2012, David K.J.; 1♂, INDIA, Tamil Nadu, Kodaikanal, 02.iv.2012, Salini S.; 1♂, INDIA, Tamil Nadu, Shenbaganur, 02.iv.2014, Veenakumari K.; 2♂♂, INDIA: Tamil Nadu, Dindigul, Thandikudi, 08.xii.2019, Sachin, K., 2♂♂ same data as above except for the collector, K.J. David; 2♂♂, 2♀♀, INDIA: Tamil Nadu, HRS Kodiakanal, 10.xii. 2019, K.J. David; 2♂♂, 3♀♀, same data as above except K. Sachin, 1 larva in slide (III instar): INDIA: Tamil Nadu, HRS Kodiakanal, 10.xii. 2019, K.J. David, (NBAIR).

**Figures 32–37.**
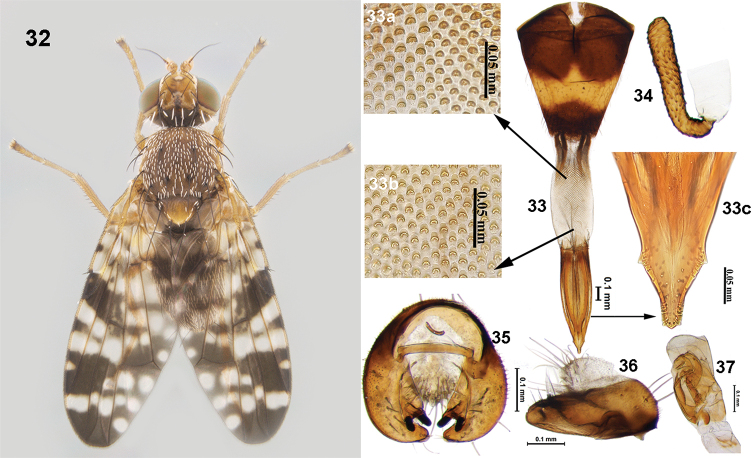
*Campiglossa gemma* (Hering) **32** habitus (dorsal view) **33** ovipositor **33a** spicules on proximal end of eversible membrane **33b** spicules on distal end of eversible membrane **33c** aculeus tip **34** spermatheca **35** epandrium and surstyli (posterior view) **36** epandrium and surstyli (lateral view) **37** glans of phallus.

###### Description.

Medium-sized fly (male 3.24–3.92 mm; female 4.49–4.83mm) with grey pollinose body, yellow legs, and reticulate wing pattern. Head slightly higher than long; frons fulvous with two frontal setae, two orbital setae (posterior orbital seta white), postocellar and postvertical seta white, lateral vertical seta white, medial vertical seta black, ocellar seta black longer than frontal and orbital seta. Scutum grey pollinose, with postpronotal lobe and notopleuron pale yellow, and well-developed chaetotaxy; posterior notopleural seta white. Scutellum with two pairs of scutellar setae; apical setae as long as basal setae. Legs fulvous, without any black markings. Wing with reticulate pattern; pterostigma black, without any hyaline spot or marking; apex of cell r_2+3_ and r_4+5_ without hyaline spot. Abdomen grey pollinose, without any markings.

***Male postabdomen***: Epandrium elongate, without clear delineation between epandrium and surstylus; lateral surstylar flange lacking, proctiger hyaline, as high as epandrium. Epandrium and surstyli oval in outline (posterior view), medial surstylus with well-developed prensisetae. Phallus 1.58 mm long, with well sclerotised glans (Fig. 37).

***Female postabdomen***: Oviscape brown (0.98 mm), with a median yellow band; eversible membrane (0.78 mm) with spicules on distal and proximal end an inverted U-shaped; distal spicules smaller compared to proximals; aculeus broad, with two preapical indentions (0.77 mm); spermatheca elongate, tubular, with striations.

###### DNA barcode.

GenBank accession number MT169785 (1♀, INDIA: Tamil Nadu, Kodaikanal HRS, 3.x.2019, K.J.David) .

###### Third instar larva (Figs 38–40).

Larva short (2.66 mm), oblong, dull creamy white, with a black triangular marking posterodorsally. Mouthhook pointed with a well-developed preapical tooth as long as apical mouthhook; ventral apodeme broader than mouthook; mandibular neck not prominent; dorsal apodeme pointed posteriorly; labial sclerite elongate; hypopharyngeal sclerite 4× longer than broad; hypopharyngeal bridge pointed posteriorly; parastomal bar reaching beyond middle of hypopharyngeal sclerite; ventral bridge of hypopharyngeal sclerite not prominent; anterior sclerite present; dorsal cornua undivided; ventral cornua with two branches. Anterior spiracle weakly sclerotised with 15 tubules. Posterior spiracle with spiracular slits oval, slightly longer than wide, devoid of transverse striations; spiracles separated by a distance more than twice length of each slit; dorsal and ventral spiracular bundle absent in specimen examined; lateral spiracular bundle with three unbranched hairs.

**Figures 38–40.**
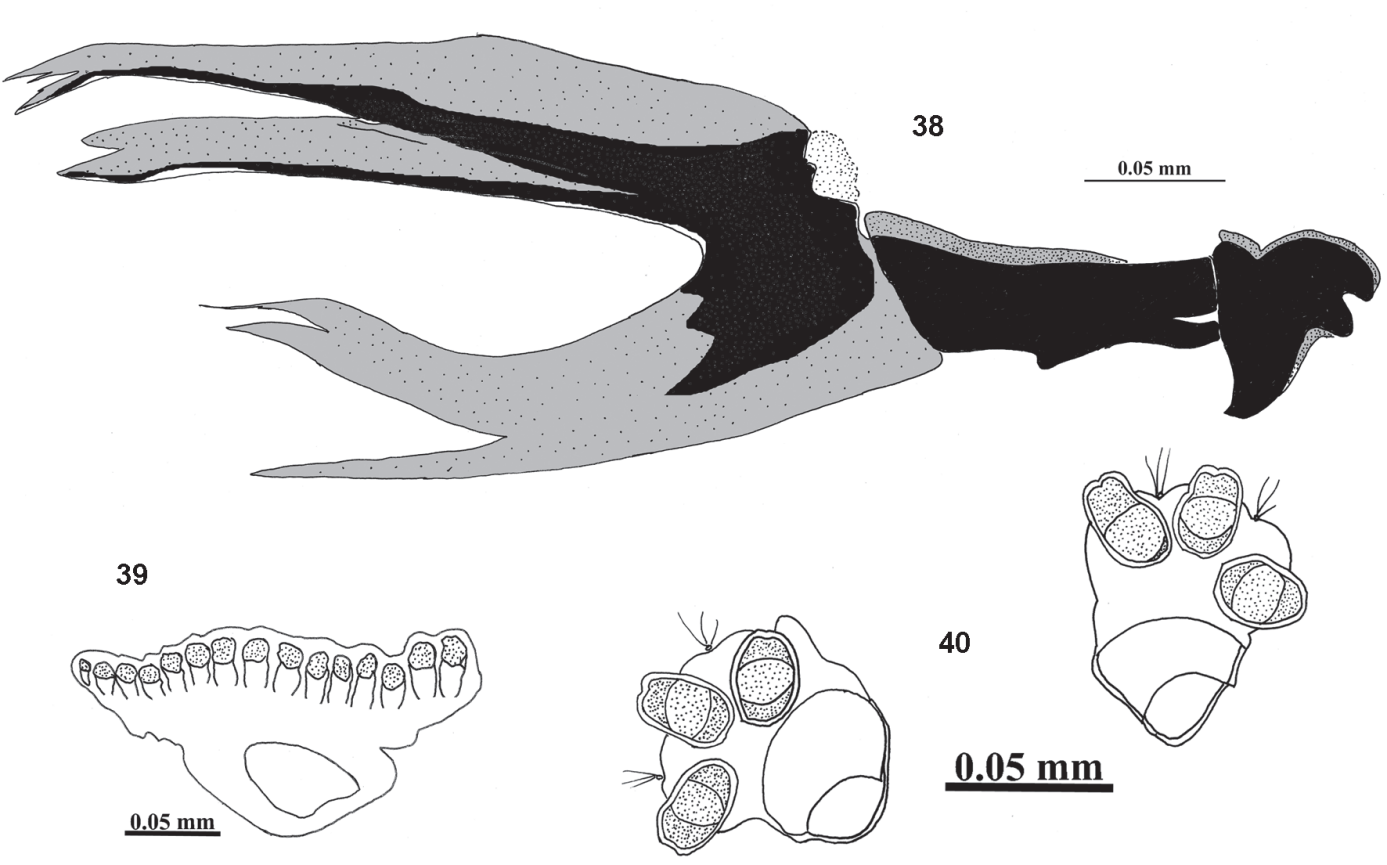
III instar larvae of *Campiglossa gemma* (Hering) **38** cephalopharyngeal skeleton **39** anterior spiracle **40** posterior spiracles.

###### Host plant.

Flowers of *Conyza* sp. (Asteraceae).

###### Remarks.

This species is known only from Tamil Nadu and western Karnataka (Kemmangundi) in southwestern India (this study; Hancock and McGuire 2002). Although there is some slight variation in wing markings, the examined specimens are consistent with Hering’s (1939) original description and most are from the type locality. In the phylogenetic tree, *C. gemma* is placed as a sister group to all the included *Campiglossa* species (Fig. 51). This might be due to the low taxon sampling or, alternatively, the species may belong to another genus, which should only be considered after a thorough study of other *Campiglossa* species and related groups.

##### Campiglossa sororcula

Taxon classificationAnimaliaDipteraTephritidae

(Wiedemann, 1830)

49A2473D-BDC9-525D-AA69-92BDA3600604

Figures 41–47

Trypeta sororcula Wiedemann, 1830: 509. Type locality: Tenerife, Canary Islands.

###### Material examined.

2♂, INDIA, Tamil Nadu, Ooty, Emerald, 17.ii.2016, Prabhu G., 1♀, INDIA, Karnataka, Bengaluru, Attur, 08.xi.2016, Prabhu G., 1♂, INDIA, Karnataka, Bengaluru, Attur, 16.v.2017, Prabhu G., 1♂1♀, INDIA, Karnataka, Bengaluru, Attur, 04.vii.2017, Prabhu G., 1♀, INDIA, Karnataka, Bengaluru, Attur, 07.viii.2018, Prabhu G., 1♀, INDIA, Karnataka, Bengaluru, Attur, 16.viii.2018, Prabhu G., 1♀, INDIA, Karnataka, Bengaluru, Attur, 21.iii.2018, Prabhu G., 2♀3♂, INDIA, Karnataka, Bengaluru, G.K.V.K, 17.vi.2019, Sachin K., 2♀2♂, INDIA, Kerala, Palakkad, Nelliyampathy, 11.xii.2019, David K.J., 3♂, INDIA, Kerala, Palakkad, Nelliyampathy, 11.xii.2019, Sachin K., 4♂, INDIA, Karnataka, Bangalore, Attur, 18.ii.2020, Maruthi K.V., 1 larva in slide (III instar): INDIA: Karnataka, Bangalore, Attur, 12.vii. 2019, Sachin, K., (NBAIR)

**Figures 41–47.**
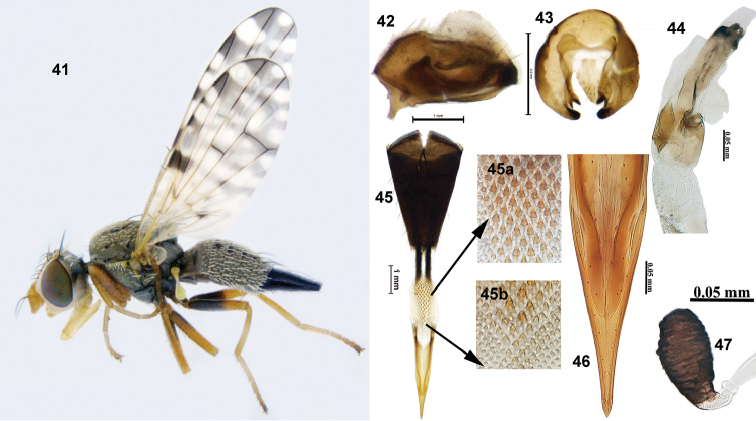
*Campiglossa sororcula* (Wiedemann) **41** habitus (lateral) **42** epandrium (lateral view) **43** epandrium (posterior view) **44** glans of phallus **45** ovipositor **45a** spicules on proximal end of eversible membrane **45b** spicules on distal end of eversible membrane **46** aculeus **47** spermatheca.

###### Description.

Small fly (male 2.37–2.94 mm; female 3.0–3.39 mm) with grey pollinose body, yellow legs, and reticulate wing pattern. Head longer than high, frons with two frontal setae, two orbital setae (posterior orbital seta white), postocellar, postvertical seta white, lateral vertical seta white, medial vertical seta black, ocellar seta black and longer than frontal and orbital seta. Scutum grey pollinose with postpronotal lobe and notopleuron pale yellow and well-developed chaetotaxy, posterior notopleural seta black. Scutellum with two scutellar setae. Legs with fulvous black patches on mid and hind femur. Wing with reticulate pattern; pterostigma black without any hyaline spot or marking; apex of cell r_4+5_ with a hyaline spot. Abdomen grey pollinose; tergites 3–5 with a pair of quadrate, submedian, black markings.

***Male postabdomen***: Epandrium elongate, without clear delineation between epandrium and surstylus; lateral surstylar flange lacking; proctiger hyaline, shorter than epandrium. Epandrium and surstyli circular in outline in posterior view; medial surstylus with well-developed prensisetae. Phallus 1.05 mm long, with well sclerotised glans (0.25 mm) (Fig. 44).

***Female postabdomen***: Oviscape black (0.81 mm); eversible membrane (0.57 mm) with spicules on distal and proximal end inverted conical; distal spicules smaller compared with proximals; aculeus pointed (0.65 mm), without preapical indentions; spermatheca oval, with striations.

###### DNA barcode.

GenBank accession number MT019889 (1♀, INDIA: Karnataka, Bangalore, Attur, 29.v.2019, K. Sachin.)

###### Third instar larva (Figs 48–50).

Larva short (3.08 mm), elongate, fusiform, creamy white. Mouthhook pointed, with a well-developed preapical tooth as long as the apical mouthhook; ventral apodeme broader than mouthook; mandibular neck not prominent; dorsal apodeme dagger-shaped, pointed posteriorly; labial sclerite elongate; hypopharyngeal sclerite 2–2.5× longer than broad; hypopharyngeal bridge pointed posteriorly; parastomal bar reaching beyond the middle of hypopharyngeal sclerite; ventral bridge of hypopharyngeal sclerite not prominent; anterior sclerite not prominent; dorsal cornua divided apically; ventral cornua with two branches. Anterior spiracle weakly sclerotised, with four tubules. Posterior spiracle with spiracular slits oval, slightly longer than wide, devoid of transverse striations; spiracles separated by a distance as equal to length of each slit; dorsal, ventral, and lateral spiracular bundle absent in specimen examined.

**Figures 48–50.**
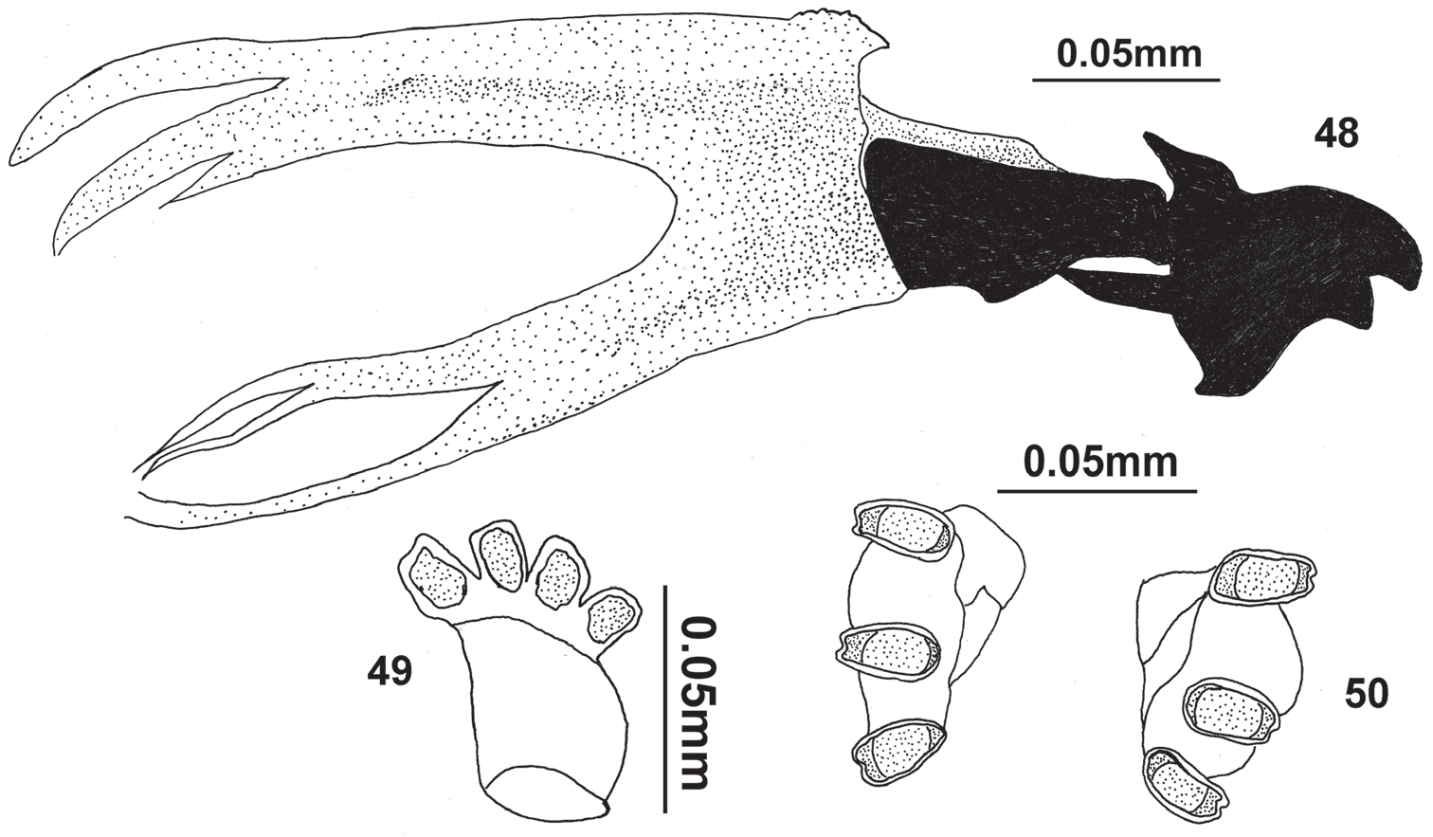
Third instar larva of *Campiglossa sororcula* (Wiedemann) **48** cephalopharyngeal skeleton **49** anterior spiracle **50** posterior spiracles.

Host plants recorded during the study: flowers of *Bidens pilosa* L. and *Cosmos sulphureus* Cav. (Asteraceae).

###### Remarks.

This species occurs commonly from southern Europe to Africa, Asia, and Australia, and has been introduced into Hawaii (Norrbom et al. 1999). Bezzi (1913), Hancock and McGuire (2002), Agarwal and Sueyoshi (2005), and David and Ramani (2011) recorded it from various locations in India, where it is widespread. Leg colour in many populations is variable (Hardy and Drew 1996); in India, the femora are generally yellow with a black basal patch on mid and hind femora. Vestigial apical scutellar setae have been observed in some Australian populations (Hardy and Drew 1996), but Indian specimens lack the apical pair.

**Figure 51.**
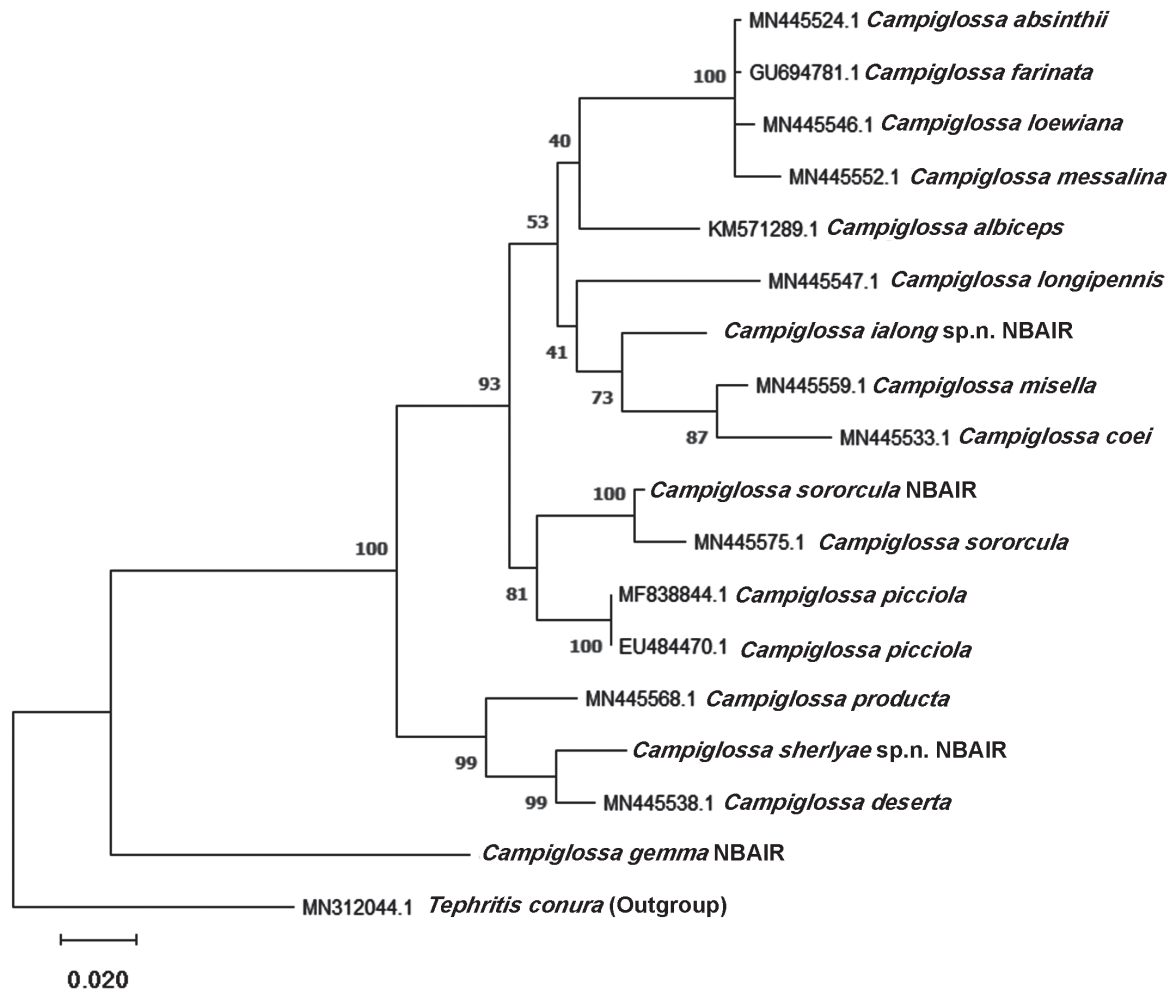
Maximum likelihood phylogram of 17 *Campiglossa* and one *Tephritis* (outgroup) DNA barcode sequences using General Time Reversible model. The number at each node is the boostsrap value based on ML analysis.

##### Campiglossa cribellata

Taxon classificationAnimaliaDipteraTephritidae

Bezzi, 1913

FCF3DD85-7E0B-5C50-8CF1-7DD6377B01A0

Campiglossa cribellata Bezzi, 1913: 161. Type locality: Kurseong, E. Himalayas, West Bengal, India.

###### Remarks.

This species belongs in the *irrorata* group and was illustrated by Bezzi (1913) and Kapoor (1993). It is known only from the eastern Himalayas in India and Nepal (Bezzi 1913; Kapoor et al. 1979b). The host plant is unknown. The holotype, deposited in ZSI, is damaged (Banerjee, D; Diptera Section, ZSI, pers. comm.) and was not available on loan; hence, a detailed diagnosis and redescription are not included here.

##### Campiglossa kumaonensis

Taxon classificationAnimaliaDipteraTephritidae

Agarwal, Grewal, Kapoor, Gupta & Sharma, 1989

6163C845-A94B-50FE-B0E4-71666BCB1760

Campiglossa kumaonensis Agarwal, Grewal, Kapoor, Gupta & Sharma, 1989: 90. Type locality: between Naini Tal and Ranikhet, Uttar Pradesh, India.

###### Remarks.

This species is provisionally included in the *irrorata* group and was illustrated by Agarwal et al. (1989) and Kapoor (1993). It is distinguished from *C. cribellata* by the reduced hyaline wing markings (particularly in the pterostigma and cell r_1_) and the more elongate wing. This species is known only from the type locality. The holotype, deposited in NPC, could not be traced and might have been lost or misplaced; hence, a diagnosis and redescription are not included here. Its unusual wing shape suggests that placement in *Campiglossa* requires confirmation.

##### Campiglossa lyncea

Taxon classificationAnimaliaDipteraTephritidae

(Bezzi, 1913)

03E51954-2442-5FD5-AC3A-723AB3AB38C0

Tephritis lyncea Bezzi, 1913: 165. Type locality: Darjeeling, E. Himalayas, West Bengal, India.

###### Remarks.

*Campiglossa lyncea* is distinguished from other Indian species by its mostly black femora, white posterior notopleural seta, two hyaline marginal spots in cell r_2+3,_ and large, often coalesced, hyaline discal spots. This species is known only from northern India and includes the record of *C. absinthii* Fabricius, 1805 from Solan, Himachal Pradesh (Agarwal and Sueyoshi 2005), which was misidentified as the synonym *C. parvula* (Loew, 1862) by Kapoor et al. (1979a) and Kapoor (1993). The illustration of *C. parvula* by Kapoor (1993) closely matches *C. lyncea* of Bezzi (1913), whereas the figure of *C. lyncea* in Kapoor’s (1993) publication is copied from Hardy (1973) and is neither this species nor Indian. Hence, Hardy’s (1973) Vietnamese records, considered to be conspecific with Kapoor’s (1993) figure of ‘*C. lyncea*’ by Hancock (2008), are also excluded. The syntypes of *C. lyncea*, deposited in ZSI, are damaged (Banerjee, D; Diptera Section, ZSI, pers. comm.) and were not available on loan. Hence, a detailed diagnosis and redescription are not included.

##### Campiglossa producta

Taxon classificationAnimaliaDipteraTephritidae

(Loew, 1844)

7456A11B-A2B9-57BC-9B60-62C3CD4614DA

Trypeta producta Loew, 1844: 399. Type locality: Turkey.

###### Remarks.

This species was recorded from India by Hancock and McGuire (2002), based on two males and two females from Gulmarg, Kashmir. However, given the complexity of this group, additional material is required for confirmation. Elsewhere, it is widespread from Western Europe to Central Asia, including Afghanistan (Agarwal and Sueyoshi 2005).

## Supplementary Material

XML Treatment for Campiglossa

XML Treatment for Campiglossa ialong

XML Treatment for Campiglossa shaktii

XML Treatment for Campiglossa sherlyae

XML Treatment for Campiglossa gemma

XML Treatment for Campiglossa sororcula

XML Treatment for Campiglossa cribellata

XML Treatment for Campiglossa kumaonensis

XML Treatment for Campiglossa lyncea

XML Treatment for Campiglossa producta
